# Supplementary material for: The Systems Biology Research Tool: evolvable open-source software
Source: BMC Syst Biol. 2008 Jun 29;2:55. doi: 10.1186/1752-0509-2-55 (PMC2446383; doi:10.1186/1752-0509-2-55)
Supplement: Additional file 1 — SBRT Archive. An archive of the current version of the Systems Biology Research Tool. [file 1752-0509-2-55-S1.zip › sbrt-1.4.0/doc/developers_guide/api/sbrt/shell/proc/algebra/package-frame.html]

sbrt.shell.proc.algebra


sbrt.shell.proc.algebra

Classes 
  
LinearCombConverterManager
  
MatrixConverterManager
  
MultipleVectorsConverterManager
  
SingleVectorConverterManager
